# Development and Validation of Lactate Metabolism-Related lncRNA Signature as a Prognostic Model for Lung Adenocarcinoma

**DOI:** 10.3389/fendo.2022.829175

**Published:** 2022-03-29

**Authors:** Shijie Mai, Liping Liang, Genghui Mai, Xiguang Liu, Dingwei Diao, Ruijun Cai, Le Liu

**Affiliations:** ^1^ Department of Thoracic Surgery, Nanfang Hospital, Southern Medical University, Guangzhou, China; ^2^ Department of Gastroenterology, State Key Laboratory of Organ Failure Research, Guangdong Provincial Key Laboratory of Gastroenterology, Nanfang Hospital, Southern Medical University, Guangzhou, China; ^3^ Department of Gastroenterology, Shenzhen Hospital, Southern Medical University, Shenzhen, China

**Keywords:** lung adenocarcinoma, lactate metabolism, immune checkpoint, lncRNA, gene signature

## Abstract

**Background:**

Lung cancer has been a prominent research focus in recent years due to its role in cancer-related fatalities globally, with lung adenocarcinoma (LUAD) being the most prevalent histological form. Nonetheless, no signature of lactate metabolism-related long non-coding RNAs (LMR-lncRNAs) has been developed for patients with LUAD. Accordingly, we aimed to develop a unique LMR-lncRNA signature to determine the prognosis of patients with LUAD.

**Method:**

The Cancer Genome Atlas (TCGA) and Gene Expression Omnibus (GEO) databases were utilized to derive the lncRNA expression patterns. Identification of LMR-lncRNAs was accomplished by analyzing the co-expression patterns between lncRNAs and LMR genes. Subsequently, the association between lncRNA levels and survival outcomes was determined to develop an effective signature. In the TCGA cohort, Cox regression was enlisted to build an innovative signature consisting of three LMR-lncRNAs, which was validated in the GEO validation cohort. GSEA and immune infiltration analysis were conducted to investigate the functional annotation of the signature and the function of each type of immune cell.

**Results:**

Fourteen differentially expressed LMR-lncRNAs were strongly correlated with the prognosis of patients with LUAD and collectively formed a new LMR-lncRNA signature. The patients could be categorized into two cohorts based on their LMR-lncRNA signatures: a low-risk and high-risk group. The overall survival of patients with LUAD in the high-risk group was considerably lower than those in the low-risk group. Using Cox regression, this signature was shown to have substantial potential as an independent prognostic factor, which was further confirmed in the GEO cohort. Moreover, the signature could anticipate survival across different groups based on stage, age, and gender, among other variables. This signature also correlated with immune cell infiltration (including B cells, neutrophils, CD4^+^ T cells, CD8^+^ T cells, etc.) as well as the immune checkpoint blockade target CTLA-4.

**Conclusion:**

We developed and verified a new LMR-lncRNA signature useful for anticipating the survival of patients with LUAD. This signature could give potentially critical insight for immunotherapy interventions in patients with LUAD.

## Introduction

Lung cancer is the most prevalent malignant tumor with ~2.1 million new cases annually and the greatest contributor to cancer-related deaths at ~1.8 million worldwide in 2018 (1, 2). In general, lung cancer can be categorized into two kinds: small cell lung cancer (SCLC) and non-small cell lung cancer (NSCLC). Lung adenocarcinoma (LUAD) and squamous cell carcinoma (SCC) of the lung are the two most common kinds of NSCLC, accounting for over 80% of all lung cancer cases. LUAD is the more common histological form with an increasing rate of occurrence (2). Recent years have seen significant advancements in lung cancer treatment, including targeted therapy and immunotherapy. LUAD has been shown to target the Kirsten rat sarcoma viral oncogene (*KRAS*) gene, the anaplastic lymphoma kinase (*ALK*) gene, and the epidermal growth factor receptor (EGFR) (3). Despite the reported efficacy of immune checkpoint blockade (ICB) as a treatment for late-stage LUAD, further research is needed to further boost their efficacy (4). Notwithstanding advancements in timely diagnosis and drug formulation, patients with advanced LUAD have dismal clinical outcomes-those with local LUAD have a five-year survival rate ranging between only 4–17% (5). Moreover, owing to the asymptomatic nature of LUAD in its early stage, the majority of patients are identified with late-stage LUAD or distant metastases (6, 7). Therefore, the identification of novel molecular markers and prognostic indicators for LUAD is imperative to enhance therapeutic outcomes and minimize the burden of disease (8).

Cancer cells with abnormal metabolic activities utilize an excessive quantity of nutrients and oxygen, leading to hypoxia, nutritional deficiencies, and increased production of harmful metabolic byproducts in the tumor microenvironment (TME) (9). Increased levels of metabolites in the TME, especially lactate, create an immunosuppressive environment favorable for cancer cell proliferation and immune evasion (10). Lactate is not only a byproduct of glycolysis but also a critical modulator in normal and malignant tissue signaling pathways (11–13). The presence of high lactate levels in tumors is associated with greater metastasis, tumor resurgence, and a dismal prognosis (11, 14). Lactate mediates cancer cells’ effects on intrinsic metabolism, as well as non-tumor cell-autonomous effects that contribute to carcinogenesis (15). Tumor cells may metabolize lactate or transfer it to surrounding cancer cells, immune cells, ivascular endothelial cells and adjoining stroma, resulting in metabolic reconfiguration (8). Furthermore, lactate is involved in tumor inflammation and acts as a signaling molecule that promotes tumor angiogenesis (16). In recent years, inhibition of lactate metabolism has proven to be a potential therapeutic approach, particularly for drug-resistant malignant tumors (17). Furthermore, various studies have shown that genes involved in lactate metabolism performed a critical function in the progression of LUAD (18–22).

Long non-coding RNAs (lncRNAs) are described as RNAs that exceed two hundred nucleotides in length. They are translated from intergenic sections or any section of the sense or anti-sense DNA strand of a protein-coding gene (23). Although lncRNAs are fundamental affiliates of the non-coding RNA family, they are not transcribed into proteins. Instead, they influence gene expression at both the translational and post-translational stages (24). The lncRNAs perform a wide range of functions such as binding proteins, targeting mRNAs for degeneration, harboring intronic miRNAs, and modulating gene expression *via* competitive endogenous RNA (ceRNA) networks (24). In cancer, dysregulation of lncRNAs is extensively prevalent and has been demonstrated to enhance carcinogenesis and progression (25). Numerous studies have investigated the lncRNA expression profile of LUAD and their potential as diagnostic and prognostic indicators of patients with LUAD (26). Lu et al., for example, used 12 pyroptosis-related lncRNAs to develop a prognostic signature for predicting LUAD patients’ 1-, 2-, 3-, 4-, and 5- year OS (27). Using 14 autophagy-related lncRNAs (ARlncRNAs), Chen et al. discovered a signature for evaluating the 1-, 3-, and 5- year survival probability in LUAD patients (28). Using three critical immune-related genes, Qi et al. developed a prognostic signature for predicting 1-, 3-, and 5- year OS (IRGs) (29). In addition, Shao et al. developed a prognostic signature based on seven hypoxia-associated lncRNAs (HRlncRNAs) for predicting 1-, 3-, and 5- year OS in LUAD patients (30). Notably, recent research has shown that lncRNAs might influence the progression of cancer by modifying the lactate metabolism (31, 32). Salmena et al. introduced the ceRNA concept, which is based on a large-scale regulatory network architecture that represents the complicated cross-talk involving coding and non-coding RNAs (33). It has been hypothesized that lncRNAs could influence lactate metabolism-related (LMR) mRNA expression *via* sponging miRNAs in the LUAD (33). Despite a few studies establishing ceRNA networks for LUAD, the modulatory mechanism of lactate-related ceRNA is still unknown (34).

Hence, the focus of the present study is to identify a new LMR-lncRNA signature able to anticipate the prognosis of patients with LUAD. By retrieving data from The Cancer Genome Atlas (TCGA) database as a training cohort, Cox regression was enlisted to build the signature consisting of three LMR-lncRNAs, which was validated using datasets from the Gene Expression Omnibus (GEO) database. This study investigated the functional enrichment of the LMR-lncRNA signature and performed a visualization of a signaling pathway that is intimately related to immunological and tumor progression. Moreover, we investigated its impact on the responsiveness to ICB treatment in patients with LUAD. The findings of this study could provide novel insight to improve existing diagnostic, treatment, follow-up, and preventive measures for patients with LUAD.

## Materials and Methods

### Collection of Datasets and Clinical Data

The RNA sequencing (RNA-seq) data, including mRNA, 1ncRNA, and miRNA expression data of LUAD samples, were downloaded from the TCGA (https://portal.gdc.cancer.gov/) database. These data were in the format of HT-seq raw read count, before log2-transformed and normalized by the “limma” package in R v3.48.3. LncRNAs and mRNAs were annotated according to the Genome Reference Consortium Human Build 38 (GRCh38) from the GENCODE websit2.Perl v5.28.1 used for data processing. As a result, clinical data of 617 LUAD samples, including survival information, age, sex, tumor stage, and TNM stage, were also downloaded from the TCGA database. We screened 535 samples according to the following inclusion criteria: (1) samples with complete overall survival (OS) and survival state; (2) samples with OS **≥**30 days; and (3) samples with complete RNA-seq data. The clinical information of 535 TCGA LUAD samples is shown in **Supplementary Table S1**. There were 951 individuals with LUAD included in the GSE72094, GSE31210, GSE30219, GSE37745, GSE50081, and GSE29013 datasets obtained from the GEO database (http://www.ncbi.nlm.nih.gov/geo/). A list of LMR genes (referring to genes included in the lactate metabolism pathway) was extracted from the map01212 gene set of the Kyoto Encyclopedia of Genes and Genomes (KEGG) database, and the expression of 245 genes was also available from the TCGA database. For the training cohort, the TCGA dataset was used; for the validation cohort, the GSE72094, GSE31210, GSE30219, GSE37745, GSE50081, and GSE29013 datasets were used.

### Data Processing for lncRNAs and LMR Genes

The “limma” R package was used to identify genes involved in lactate metabolism that exhibited differential expression, and the outcomes were depicted with heatmaps and volcano plots. To discover the key biological features, we conducted a functional enrichment analysis of the data using Gene Ontology (GO) and KEGG. The “GOplot” R program was used to provide a visual representation of the enrichment terms.

The Pearson correlation was utilized to identify the relationship between candidate LMR-lncRNAs and LMR genes with differential expression. In this study, LMR-lncRNAs were defined as those with *P <*0.001 and |*R* ^2^| >0.3. Lastly, the co-expression network of predictive lncRNAs and LMR genes was generated using Cytoscape v3.8.2.

### Development of Prognostic LMR-lncRNA Signature

First, the LMR-lncRNAs related to prognosis were identified in the training cohort utilizing univariate Cox regression. Next, to construct the LMR-lncRNA signature, LMR-lncRNAs with *P* ≤0.05 were included in the multivariate Cox regression. The equation used in the present study was as follows: Risk score of LMR-lncRNA signature = $\Sigma_{i}^{n}\;Coefficient\;(LMR-lncRNAi)\times Expression\;(LMR-lncRNAi)$ . With the aid of a receiver operating characteristic (ROC) curve at one year for OS, the optimal threshold value of the LMR-lncRNA signature was determined to categorize the patients into high- and low-risk groups. The Kaplan-Meier (KM) approach was utilized to examine the survival rate between the two risk groups, whereas the log-rank test was utilized to compare the outcomes.

The nomogram was employed to anticipate the survival of patients with LUAD over the course of 1-, 3-, and 5- years. To determine the accuracy of the model based on the training cohort, ROC and calibration curves were utilized. Subsequently, to determine the reliability of the LMR-lncRNA signature as an independent predictor of prognosis in patients with LUAD, we adjusted other clinical characteristics (i.e., smoking history, stage, gender, age) in an independent prognostic analysis.

### Verification of the LMR-lncRNA Signature

To test the robustness of the model, the GEO database was enlisted in the training cohort. The LMR-lncRNA signature was derived using the validation cohort. Subsequently, in the validation cohort, Cox regression and survival analysis were employed to determine whether the LMR-lncRNA signature was substantially correlated with OS. The ROC curves were visualized to determine if the innovative model might effectively anticipate the survival of patients with LUAD.

### Nomogram Development and Gene Set Variation Analysis (GSVA)

A nomogram was developed based on the risk score and other clinical indicators to predict the 1-, 3-, and 5-year OS of patients with LUAD. We used calibration curves for the data to determine the nomogram’s predictive accuracy. The GSVA was used to see if there were any functional phenotypic differences between the low- and high- risk groups (https://www.bioconductor.org/packages/release/bioc/html/GSVA.html). The reference gene sets in this study were h.all.v7.2.symbols.gmt [cancer hallmarks].

### The Value of the LMR-lncRNA Signature in Clinical Care Management

To predict the relative levels of tumor-infiltrating immune cells (TIICs) across the two risk groups based on the LMR-lncRNA signature, we employed algorithms that included the single-sample GSEA (ssGSEA) (35), TIMER (36), QUANTISEQ (37), MCP counter (38), ESTIMATE (39), EPIC (40), and CIBERSORT (41). When comparing the concentration of TIICs under various algorithms, a heatmap was used to depict the differences. Furthermore, a correlation analysis between the LMR-lncRNA signature and TIICs abundance was conducted to determine the possible influence of the LMR-lncRNA signature on the immune characteristics based on the findings of TIMER. The Wilcoxon signed-rank test using pRRophetic and ggplot2 in R was used to assess the half inhibitory concentration (IC50) values for several anticancer medications suggested for LUAD treatment, such as cisplatin, docetaxel, erlotinib, paclitaxel, gemcitabine, and gefitinib.

### Statistical Analysis

All statistical analyses were performed using R programming language v3.6.1. To compare the differences in the survival of patients between the two risk groups, the KM analysis combined with the log-rank test from the “survival” R package was utilized. Multivariate and univariate analyses were performed utilizing the Cox proportional hazards regression model to determine the prognostic significance of the LMR-lncRNA signature. The implementation of stratification analysis was accomplished based on gender (female and male), age (**≥**65 and <65 years), and stage (stages 1-2 and 3-4). To distinguish between the two risk groups of functional annotations, GSEA was conducted. Statistical tests were bilateral and a *P*-value <0.05 indicated a significant difference.

## Results

### Differential Expression of LMR-lncRNAs

**Figure 1:**
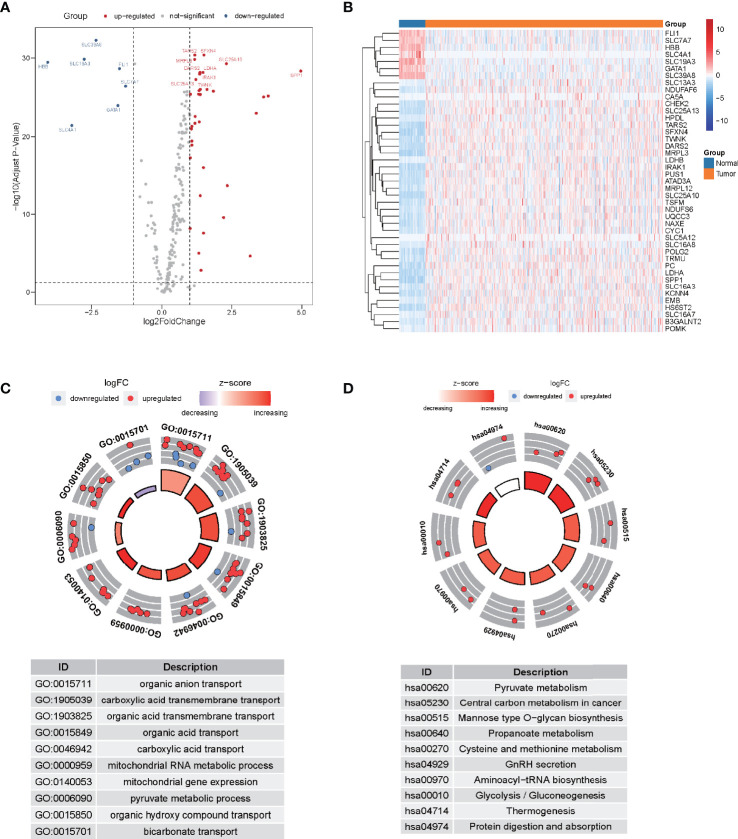
The differential expression of lactate metabolism-related (LMR)-lncRNAs. **(A)** Volcano plot of differentially expressed genes associated with lactate metabolism. Red dots denote up-modulated genes, while the blue dots down-modulated genes. The plot shows the top gene symbols with the most variance. **(B)** Heatmap of the expression of 43 LMR genes with a significant degree of variance in the training cohort. **(C)** Circle plots of functional enrichment in GO. Red dots denote up-modulation, while the blue dots down-modulation. **(D)** Circle plot of KEGG enrichment analysis. The Z-score is strongly correlated with the enrichment levels.

Following the extraction of 262 LMR gene expression data from the TCGA database (of samples from patients with LUAD), 69 genes were found to be up-modulated and 13 genes were found to be down-modulated (false discovery rate [FDR] <0.05, log2fold-change [FC] >1). Volcano plot and heatmap plot were utilized to show the genes correlated with lactate metabolism that had differential expression (**Figures 1A, B**). Following the GO enrichment analysis, it was shown that the LMR genes that were differentially expressed predominantly performed a function in the pyruvate metabolic process, carboxylic acid transmembrane transport, and organic anion transport (**Figure 1C**). The key KEGG pathways included some cancer-related signaling pathways, pyruvate metabolism, thermogenesis, and glycolysis/gluconeogenesis (**Figure 1D**).

### Detection of Prognostic LMR-lncRNAs for the Signature

**Figure 2:**
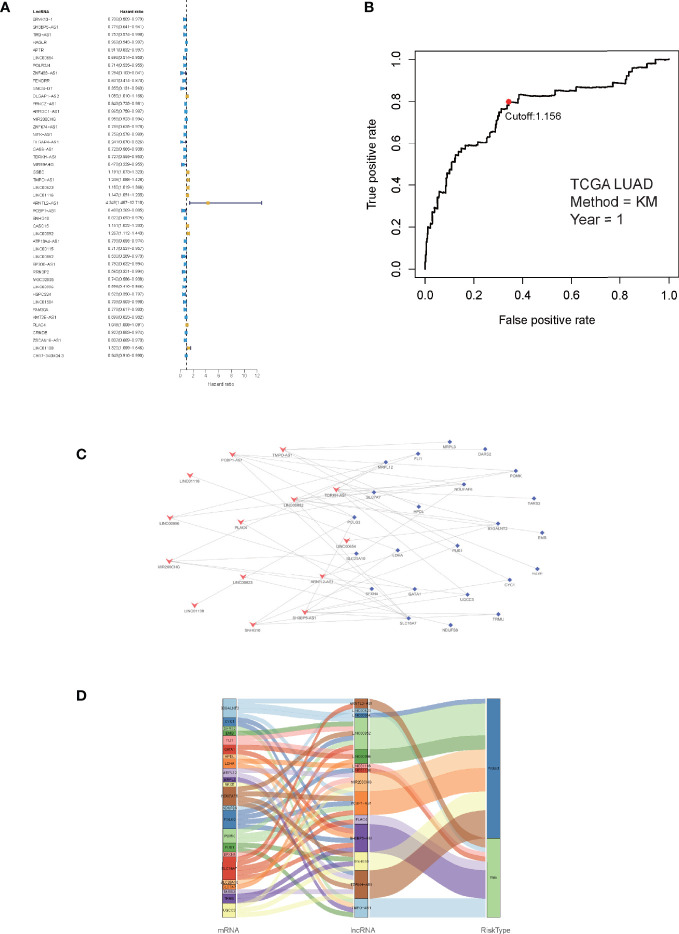
Identification of prognostic lactate metabolism-related (LMR)-lncRNAs. **(A)** Forest plots of LMR-lncRNAs associated with prognosis based on univariate Cox regression findings. **(B)** ROC curve after one year for the signature of LMR-lncRNAs in the training group. The threshold value was 1.038, which was used to divide patients into high- and low-risk groups. **(C)** The network of relationships between signature and relevant co-expressed LMR genes. **(D)** The Sankey diagram of the relationship between LMR genes, prognostic LMR-lncRNAs, and risk category.

Correlation analyses were performed on the training cohort to identify lncRNAs associated with differentially expressed LMR genes. In total, a sum of 2,843 LMR-lncRNAs was discovered (|*R* ^2^| >0.3 and *P <*0.001). Among them, 380 LMR-lncRNAs were found to coexist in both the training and validation cohorts, which were then employed for the subsequent model development and verification. The combat utility in the “sva” R package was utilized to adjust the batch impacts from various cohorts. To filter prognostic-related LMR-lncRNAs, Cox regression was employed. According to the findings of the univariate Cox analysis, 45 LMR-lncRNAs exhibited prognostic significance for patients with LUAD **(** *P <*0.05, **Figure 2A**).

Furthermore, the multivariate Cox regression analysis indicated that 14 LMR-lncRNAs were correlated with the grim prognosis in patients with LUAD. These 14 LMR-lncRNAs were selected to construct a signature for LMR-lncRNAs. The following equation was utilized to compute the risk score:


$$\begin{array}{l} {\text{Risk score}}_{{\text{LMR}}_{\text{lncRNA}}\text{signature}}=(0.240244843\times SH3BP5\_AS1)+(-0.586623713\times LINC00654)\\ \;+(-0.061821949\times MIR200CHG)+(-0.290824103\times TDRKH\_AS1)+(0.347585347\times TMPO\_AS1)\\ \;+(0.158225668\times LINC00623)+(0.053975718\times LINC01116)+(1.454856534\times ARNTL2\_AS1)\\ \;+(-0.933356748\times PCBP1\_AS1)+(-0.162168901\times SNHG10)+(-0.557979693\times LINC00852)\\ \;+(-0.473801571\times LINC00996)+(0.065036293\times PLAC4)+(0.389928706\times LINC01138) \end{array}$$

**Figure 3:**
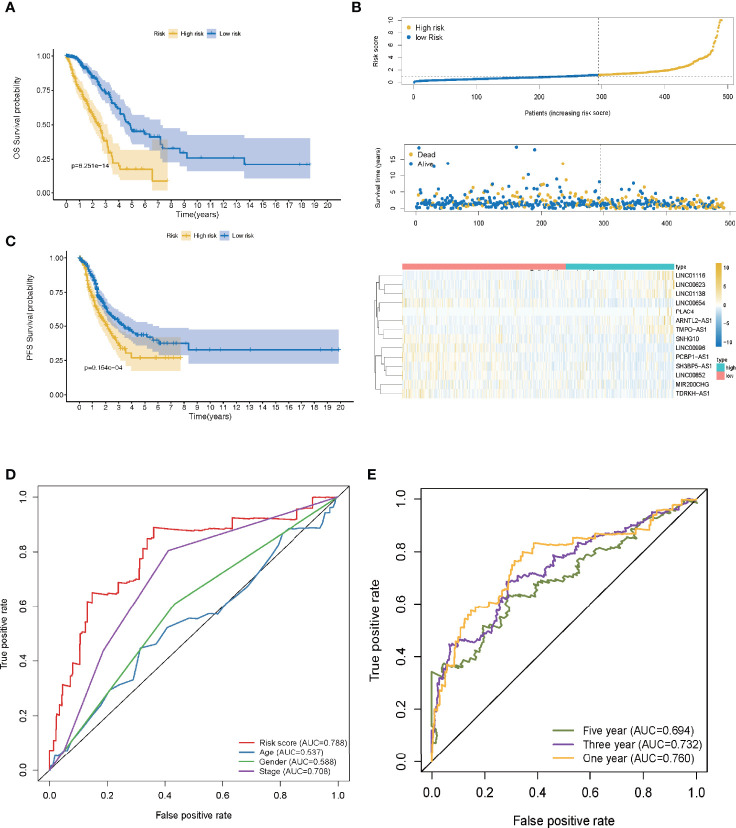
The lactate metabolism-related (LMR)-lncRNA signature based on the training cohort. **(A)** Overall survival (OS) curves for LUAD in low- and high-risk groups in the training cohort using the Kaplan-Meier method. **(B)** Distribution of patients in TCGA cohort based on the median risk score and survival status for each case. **(C)** Progression-free survival (PFS) risk profile for risk score-based patients. **(D)** The AUC values of the risk score and other clinical parameters. **(E)** 1-, 3-, and 5- year ROC curves to assess the prognostic accuracy of the LMR-lncRNA signature developed by the training cohort.

A ROC curve was utilized and the optimum threshold value for risk score was determined as 1.156. Using this threshold value, the patients with LUAD were divided into low- and high-risk groups (**Figure 2B**). As demonstrated in **Figures 2C, D**, there was a correlation between the 14 prognostic LMR-lncRNAs and the co-expressed mRNAs. The risk score was shown to be strongly associated with the OS of patients, with patients in the high-risk group exhibiting a considerably shorter OS as opposed to those in the low-risk group (*P <*0.001, **Figure 3A**). According to K-M survival analysis, PFS rates were significantly lower in the high-score group than in the low-score group (**Figure 3C**). Moreover, the area under the ROC (AUC) curve of the LMR-lncRNA signature was discovered as 0.788, indicating superior predictive performance in anticipating the prognosis of patients with LUAD compared with other conventional clinical-pathological characteristics (**Figure 3D**). In addition, the survival plot revealed that the risk score of patients was negatively associated with survival. Furthermore, the risk heatmap revealed that the levels of 14 LMR-lncRNAs correlated positively with the risk scores (**Figure 3B**). The results of the AUC for 1-, 3-, and 5- years were 0.760, 0.732, and 0.694, respectively (**Figure 3E**).

### LMR-lncRNA Signature Was an Independent Prognostic Marker

**Figure 4:**
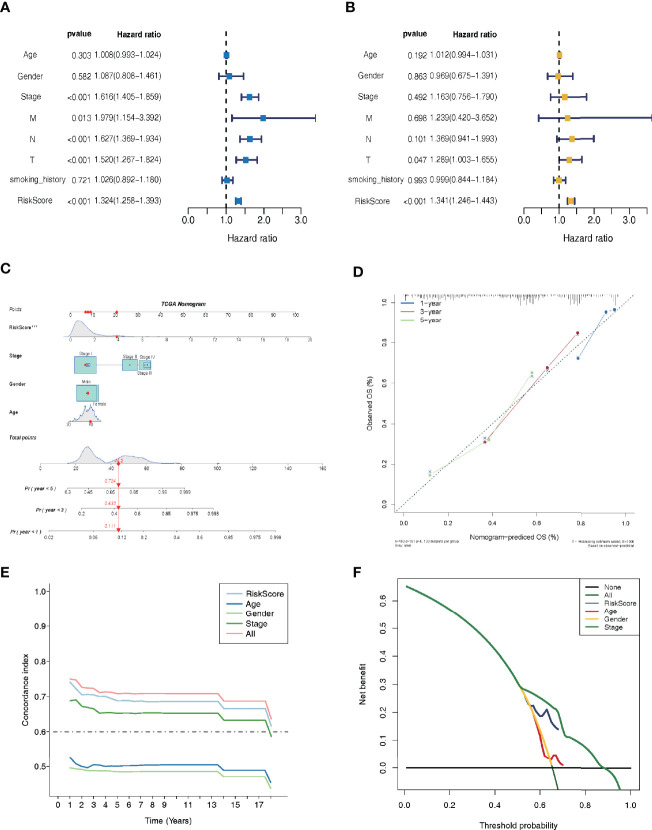
Lactate metabolism-related (LMR)-lncRNA signature for the training cohort as an independent predictive predictor with potential therapeutic significance. Cox regression analysis using univariate **(A)** and multivariate **(B)** variables to anticipate survival of patients using the LMR-lncRNA signature. **(C)** A nomogram based on clinical characteristics (e.g., the risk score) and patient survival. **(D)** The calibration chart to determine the consistency of the anticipated and actual 1-, 3-, and 5- year survival rates. **(E)** The concordance index (C-index) was determined. **(F)** Decision curve analysis (DCA) of the nomogram prediction model and the TNM staging system.

Independent prognostic factor analysis utilizing univariate data demonstrated that risk score was a prognostic marker that strongly correlated with the dismal survival (HR = 1.325, 95% CI [1.258-1.396]; *P <*0.001) (**Figure 4A**). Additionally, even though we adjusted for other clinical factors, including smoking history, stage, gender, and age, our signature remained a significant independent predictive predictor in multivariate independent analysis [HR = 1.280, 95% CI (1.214-1.350); *P <*0.001] (**Figure 4B**). As illustrated by the nomogram, the 14-LMR lncRNAs-based signature contributed the most to OS of each period in LUAD (**Figure 4C**). The curve for calibration demonstrated that the signature for LMR-lncRNAs was highly accurate (**Figure 4D**). Moreover, the concordance index (C-index) was also calculated to determine the nomogram’s ability to distinguish and predict. The C-index ranged from 0.5 to 1.0, with the higher the C-index, the better the predictive model’s distinguishing ability (**Figure 4E**). And the decision curve analysis (DCA) revealed that the nomogram outperformed the traditional TNM stage system in predicting LUAD patients survival for 5-year overall survival rates (**Figure 4F**).

### Stratification Analyses

**Figure 5:**
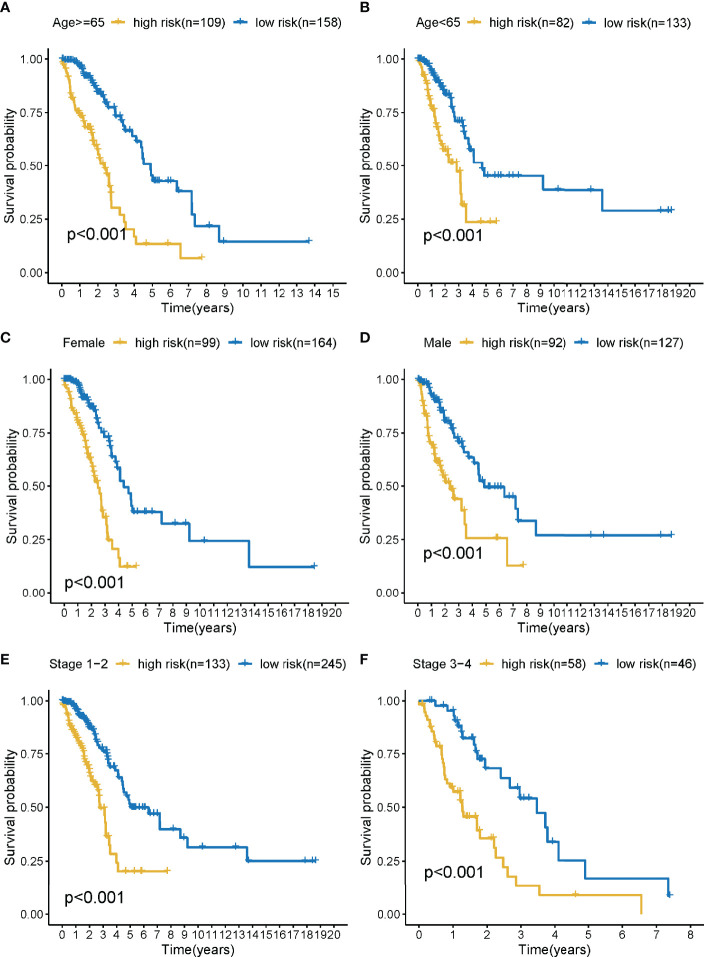
The survival curves of the LMR-lncRNA signature stratified by age, gender, and stage. **(A)** ≥65 years, **(B)** <65 years, **(C)** female, **(D)** male, **(E)** stage 1-2, and **(F)** stage 3-4.

The training cohort was subject to stratification analysis according to the clinical and pathological characteristics of LUAD (such as stage, gender, and age, etc.). Therefore, the LMR-lncRNA signature still demonstrated a strong correlation with unfavorable survival in male patients aged 65 years or above and those aged below 65 years, with early stages 1-2 or late stages 3-4 (all *P <*0.001; **Figures 5A–F**), which implied that the LMR-lncRNA signature based on the risk classification may function as a valuable tool for anticipating LUAD survival based on the stage of disease, age, and gender.

### Verification of the LMR-lncRNA Signature

**Figure 6:**
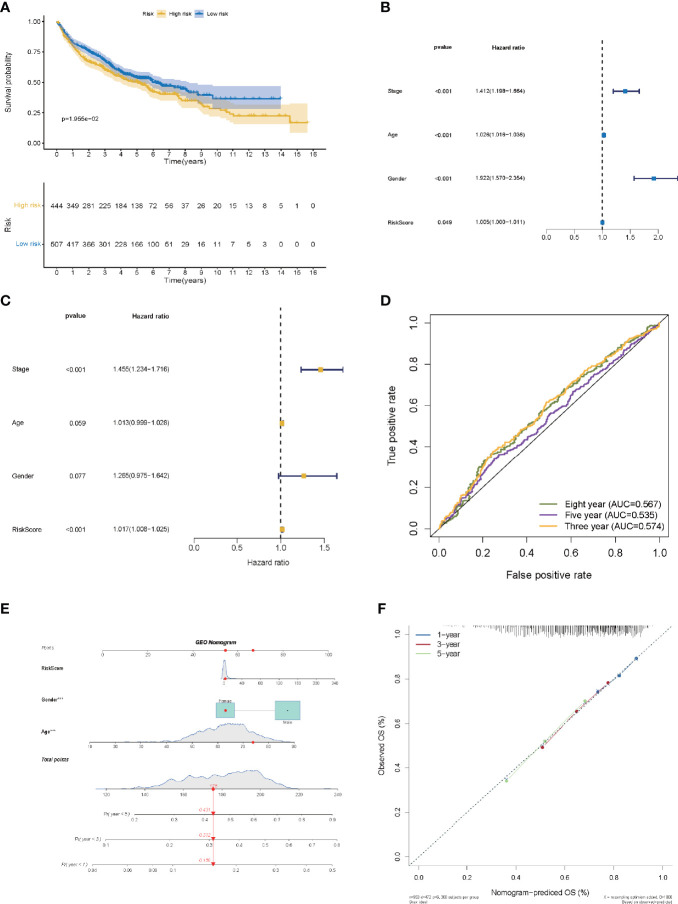
The validation of a prognostic signature of LMR-lncRNAs in a validation cohort. **(A)** The KM curves of the validation cohort demonstrated significant differences in OS between the low- and high- risk groups (*P <*0.05). COX regressions for the LMR-lncRNA signature developed from the training cohort are shown in univariate **(B)** and multivariate **(C)** analyses. **(D)** The AUC of ROC curves corroborated the prediction performance levels of the LMR-lncRNA signature in the validation cohort. **(E)** A nomogram based on clinical characteristics (e.g., the risk score) and patient survival. **(F)** The calibration chart to determine the consistency of the anticipated and actual 1-, 3-, and 5- year survival rates.

The risk score of the LMR-lncRNA signature was determined using the aforementioned equation in the validation cohort. The validation cohort’s risk score threshold value was the same as that employed in the training cohort (Cutoff = 1.156). Additionally, the signature was strongly correlated with the OS of patients with LUAD (*P <*0.05, **Figure 6A**). Independent prognostic factor analysis using univariate data demonstrated that the LMR-lncRNA signature served as a significant independent prognostic marker [HR of risk score = 1.005, 95% CI (1.000–1.011), *P <*0.05, **Supplementary Table S2** and **Figure 6B**]. Remarkably, even adjusting for gender, age, and TNM stage, the LMR-lncRNA signature remained a prognostic predictor in multivariate analysis [HR = 1.017, 95% CI (1.008-1.025), P <0.001], indicating the need for additional independent cohorts for verification (**Supplementary Table S2** and **Figure 6C**). At 3-, 5-, and 8- years, the AUC values exceeded 0.50, indicating that the LMR-lncRNA signature developed from the training cohort was highly accurate and robust (**Figure 6D**). As illustrated by the nomogram, the 14-LMR lncRNAs-based signature contributed the most to OS of each period in LUAD (**Figure 6E**). The curve for calibration demonstrated that the signature for LMR-lncRNAs was relatively accurate (**Figure 6F**).

### LMR-lncRNA Signature Regulated MYC Targets, Glycolysis, Hypoxia, and PI3K AKT mTOR Signaling

**Figure 7:**
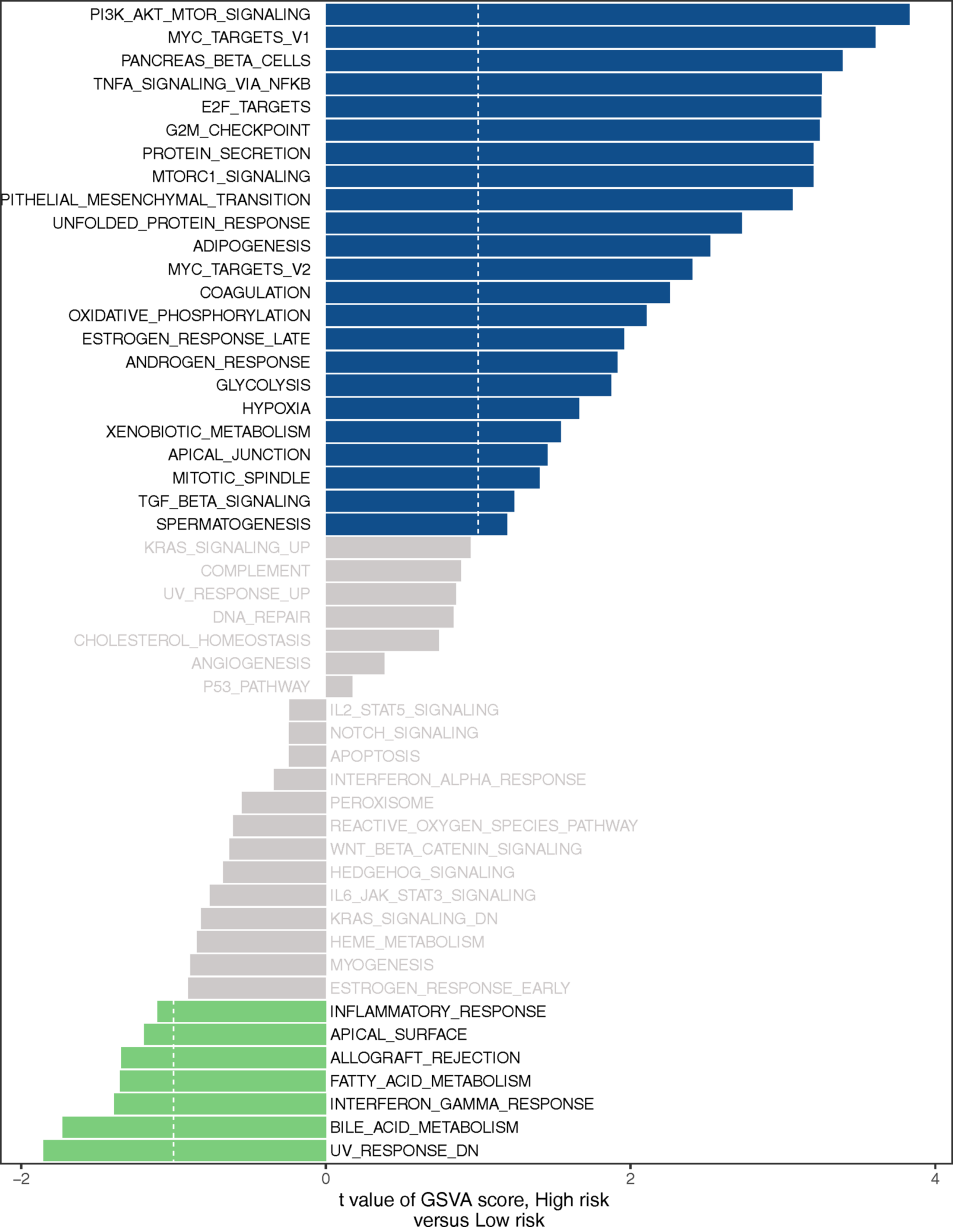
Certain cancer-related and immunologic characteristics modulated by the LMR-lncRNA signature.

GSEA was used to investigate the biological processes behind the role of the LMR-lncRNA signature in LUAD progression. The findings from cancer hallmarks analysis illustrated that the LMR-lncRNA signature in the high-risk group triggered MYC targets, glycolysis, hypoxia, and PI3K/AKT/mTOR signaling. In contrast, the LMR-lncRNA signature in the low-risk group triggered the metabolism of fatty acid, interferon-gamma response, metabolism of bile acid, and inflammatory response (**Figure 7**).

### Association of LMR-lncRNA Signature With ICB Molecules, TIICs, Immunotherapy, Chemotherapy, and Targeted Therapy

**Figure 8:**
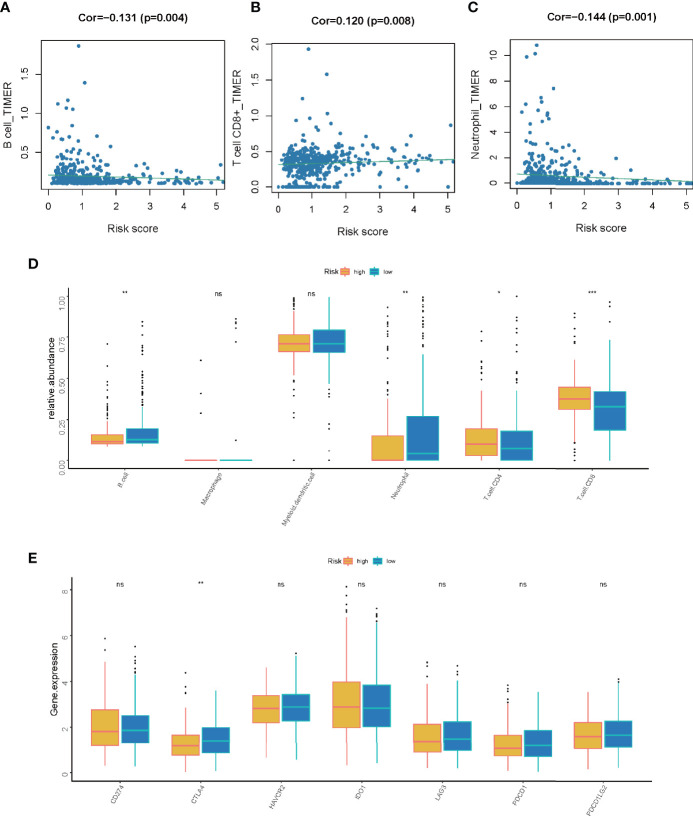
The correlation between the LMR-lncRNA signature and ICB molecules and TIICs determined using TIMER findings. **(A–C)** Relationship between the signature and the B cell **(A)**, CD8+ T cell **(B)**, or neutrophil **(C)** Spearman correlation. **(D)** The expression of *CD274*, *CTLA-4*, *HAVCR2*, *IDO1*, *LAG-3*, *PDCD1*, and *PDCD1LG2* were compared between low- and high-risk groups. **(E)** Significant and positive correlation between the LMR-lncRNA signature and the ICB receptor CTLA-4. ns, not significant; *P < 0.05; **P < 0.01; ***P < 0.001.

Based on our previous findings, the LMR-lncRNA signature was shown to modulate a wide range of immunological characteristics. As a consequence and relying on the TIMER findings, we additionally examined if the signature was related to TIICs. The findings revealed that this signature had the most positive and significant association with immunological infiltration of neutrophil cells (*R* =-0.144, *P* =0.001), CD8+ T cells (*R* = 0.120, *P* =0.008), and B cells (*R* = -0.131, *P* =0.004) (**Figures 8A–D**). The results also illustrated that the LMR-lncRNA signature was correlated with immune checkpoint inhibitors (i.e., CTLA-4), which implied that the signature has a promising potential in assessing the responsiveness to ICB treatment (**Figure 8E**). **Supplementary Figure S1** depicts a heatmap of immune responses based on ssGSEA, TIMER, QUANTISEQ, MCP counter, ESTIMATE, EPIC, and CIBERSORT. These results provided convincing evidence that the LMR-lncRNA signature was correlated with immune cell infiltration in LUAD. The TIDE system was used to generate TIDE score, LMR-lncRNA signature, T-cell dysfunction score, and T-cell exclusion score. We investigated the relationship between this risk signature and TIDE in order to further investigate the value of this model in tumor immunotherapy. In our study, high-risk patients had a higher T-cell exclusion score (**Supplementary Figure S2B**) than low-risk patients, as well as a lower TIDE score (**Supplementary Figure S2A**) and T-cell dysfunction score (**Supplementary Figure S2C**). As described in **Supplementary Figure S2D**, the relative possibility to respond to CTLA4 blocker treatment was lower in the high-risk group. All these results suggested that risk score may be related to immunotherapy. Moreover, we calculated the half-maximal inhibitory concentration (IC50) of 6 common targeted therapeutics and chemotherapeutics agents for LUAD using the pRRophetic technique to investigate the relationship between riskscore and chemoresistance. Patients with low-risk scores were extremely sensitive to the targeted therapeutic erlotinib (P =0.021), as evidenced by the half inhibitory concentration (IC50). Furthermore, patients with low risk scores were highly sensitive to the chemotherapeutics cisplatin (P = 0.039), docetaxel (P = 0.0011), and paclitaxel (P=0.00079), implying that our risk model could be used to predict chemosensitivity and guide the use of targeted therapeutics and chemotherapeutics in the clinic (**Supplementary Figure S3**).

### Analysis of Mutations

**Figure 9:**
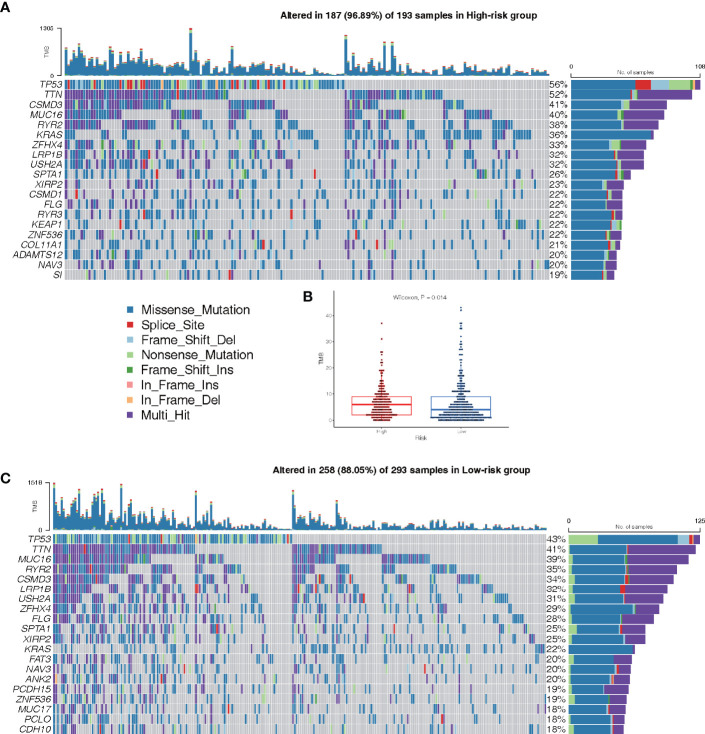
Landscape of Mutation. **(A)** The waterfall chart shows the mutation distribution of patients in the high-risk group. **(C)** The waterfall chart shows the mutation distribution of patients in the low-risk group. The right panel of the waterfall plot shows the mutation frequency, and the genes are sorted by their mutation frequency. The figure on the right shows the type of mutation. The histogram above is the statistical data of TMB of each sample. **(B)** TMB difference between high- and low-risk groups.

The mutations in the top 20 most frequently occurring genes in 193 and 293 samples from the high-risk and low-risk groups, respectively, are shown in **Figures 9A, C**. In the high-risk group, the TP53 mutation was found to be more common than in the low-risk group. Patients in the high-risk group also had more KRAS mutations than patients in the low-risk group (**Figures 9A, C**). The TMB of patients in the low-risk group was shown to be significantly lower in the box plot (**Figure 9B**).

## Discussion

At present, lncRNA signatures have been associated with glycolysis, fatty acid metabolism, and amino acid metabolism in various malignancies. Several investigations have shown that lncRNAs perform an integral function in the prognosis of patients with LUAD (42, 43), suggesting that they can serve as promising molecular targets in treating LUAD. Previous research also showed lncRNAs are implicated in multiple biological activities, including metabolism, inflammation, autophagy, and immune response (44). Furthermore, current research suggests that certain lncRNAs may perform important roles in the modulation of the occurrence and progression of illness through boosting lactate metabolism (45). Thus, LMR-lncRNAs have the potential to act as innovative molecular indicators and possible therapeutic targets in cancer therapy. However, no research has been published on an LMR-lncRNA signature as a prognostic indicator in tumors including LUAD.

As part of our investigation, we used a training cohort as well as an independent validation cohort to generate a unique LMR-lncRNA signature to anticipate the survival of patients with LUAD. The findings revealed that the LMR-lncRNA signature was primarily involved in MYC targets, glycolysis, hypoxia, and tumor-related signaling pathways. It was shown that patients in the high-risk group exhibited a shorter OS time. Furthermore, this LMR-lncRNA signature was shown to be reliable for anticipating the prognosis of patients with LUAD (high average AUC >0.788). Notably, our findings illustrated that the novel LMR-lncRNA signature had a strong predictive power for anticipating OS in individuals suffering from LUAD. The results of the stratification study demonstrated that the LMR-lncRNA signature based on the risk groups retained a high level of predictive power for anticipating survival in various indicators, such as age (<65 or **≥**65 years), stage (stages I-II or III-IV), and gender (male or female). Lastly, this signature could also act as an indicator to monitor the responsiveness of patients with LUAD to ICB immunotherapy. Hence, we generated an LMR-lncRNA signature that has excellent prognostic and predictive utility for lung cancer.

The emergence of immune checkpoint inhibitors has resulted in the creation of an innovative and promising therapeutic technique for LUAD known as ICB immunotherapy. However, only ~20% of patients with lung cancer are responsive to ICB therapy (46). Accumulating research has indicated that combining glycolytic cancer cells with cancer-associated fibroblasts elevates the levels of lactate in the TME. These high lactate levels have the potential to influence immunological responses to sustain immunosuppression in the TME through both lactate-mediated and H^+^-mediated mechanisms. Therefore, therapies aimed at avoiding such an increase are considered promising treatment options for malignancies that have been reprogrammed (47). Nonetheless, owing to its off-target impacts, lactate-targeted monotherapy has only minimal therapeutic effectiveness. Hence, integrating therapies aimed at targeting lactate along with other therapies, such as anti-VEGF therapy, anti-HER-2 therapy, anti-CTLA-4 therapy, anti-PD-1/PD-L1 therapy, ALK inhibitors, EGFR inhibitors, and mTOR inhibitors, could be an alternative treatment approach. For instance, preclinical studies have demonstrated that integrating the MCT1 inhibitor AZD3965 and anti-PD-1 treatment suppresses the infiltration of excessive PD-1^+^ Tim-3^+^ T cells in solid tumors while simultaneously improving antitumor immunity (48). In another preclinical investigation, the combination of angiogenesis inhibitors (which block the VEGF signaling pathway) and AZD3965 decreases tumor development while simultaneously decreasing both hypoxia and blood perfusion in tumor tissues (49). Despite limited research available on the combined application of tyrosine kinase inhibitors (TKIs) and MCT1 inhibitors, one preclinical study illustrated that AZD3965 strongly impedes cell proliferation and motility in both TKI-resistant and -sensitive NSCLC cells-implicating excellent potential of combination therapies to treat this disease (50). In the present study, the developed LMR-lncRNA signature was used to examine the correlation between lactate metabolism and ICIs to anticipate responsiveness to ICB immunotherapy. The results illustrated that the LMR-lncRNA signature was correlated with ICIs (i.e., CTLA-4) indicating that the signature has potential in assessing the responsiveness to ICB treatment. Furthermore, the levels of these ICIs were shown to be greater in the high-risk group as opposed to the low-risk group. This shows that the LMR-lncRNA signature might be used to anticipate ICI levels to provide valuable guidance for ICB immunotherapy efforts. Furthermore, the LMR-lncRNA signature was associated with TIICs (B cells, neutrophils, and CD8^+^ T cells) in LUAD, thereby useful to assess immunological infiltration. These results agreed with prior research demonstrating that numerous lncRNAs functioned as modulators of tumor immunology, such as TIICs and antigen release (51, 52).

Earlier research has demonstrated that lncRNAs are involved in diverse biological mechanisms, such as metabolism (52), DNA repair and cell cycle (53), and immune modulation (52, 53) amongst others. Concerning the specific LMR-lncRNA signature (*SH3BP5-AS1, LINC00654, MIR200CHG, TDRKH-AS1, TMPO-AS1, LINC00623, LINC01116, ARNTL2-AS1, PCBP1-AS1, SNHG10, LINC00852, LINC00996, PLAC4, and LINC01138*) in this study, various RNA genes are important role players in cancer progression, differentiation, and migration.

For instance, *SH3BP5-AS1* is a proven independent prognostic marker of head and neck squamous cell carcinoma (54), and upregulation of LINC00654 has been correlated with dismal OS in breast cancer (55). *MIR200CHG* can stimulate proliferation, invasion, and drug resistance in breast cancer by interacting with and normalizing YB-1, which is involved in regulating hypoxia-dependent gene transcription, and the hypo-phosphorylation of lactate metabolism related-signaling such as mTOR and HIF1 in cancer (56–58). Up-regulation of *TDRKH-AS1* is strongly associated with malignant features of colorectal cancer as well as their unfavorable prognoses (59, 60), and *TMPO-AS1* silencing reduced cancer cells proliferation and stemness while promoting apoptosis in CRC cells (61). Oncogenic *TMPO-AS1* was also abnormally up-regulated in retinoblastoma tissues which served as ceRNA to modulate the expression of HIF-1α, a core hub regulating cellular lactate metabolism by sponging miR199a-5p (62). *LINC00623* has been shown to significantly correlate with OS as a potential new molecular biomarker and performs critical functions in the modulation of hormone-related cancer progression (63). Due to clinical importance and association with biological processes in many malignancies, *LINC01116* could be a crucial biomarker for the prediction and therapeutic interventions of malignant tumors (64). *PCBP1-AS1* could facilitate the AR/AR-V7 deubiquitination, resulting in the protection of AR/AR-V7 against degeneration (65). *In vitro* and *in vivo* studies have shown that targeting PCBP1-AS1 may improve the therapeutic responsiveness of enzalutamide-resistant cancers and suppress the metastasis of LUAD (65, 66). *SNHG10* was shown to be overexpressed in gastric cancer (GC) cells. When *SNHG10* was knocked down, the proliferation, migration, and invasion of GC cells were suppressed (67). *SNHG10* was also reported to regulate glucose uptake and lactate production *via* increasing the methylation of the *miR-218* gene in osteosarcoma (68). According to one study, the expression of *LINC00852* was highly up-modulated in ovarian cancer tissues, and it functioned as a ceRNA of miR-140-3p, promoting the expression of *AGTR1* and activating the MEK/ERK/STAT3 signaling pathways. The proliferation and invasion of ovarian cancer cells were suppressed *in vivo* after the knockdown of *LINC00852* (69). The reduced expression of *LINC00996* may engage in CRC progression and metastasis, and depleting LINC00996 is related to a grim prognosis in patients with CRC. Furthermore, the HIF1 pathway was one of the major pathways modulated by *LINC00996* in the progression of CRC (70). HIF1 plays a key role in the reprogramming of cancer metabolism by activating transcription of genes encoding glucose transporters and glycolytic enzymes, which take up glucose and convert it to lactate (71). Severe early-onset pre-eclampsia is one of the most serious complications of pregnancy. *PLAC4* is a gene that is significantly expressed in the placenta compared with other tissues in the body. In a recent test for its biomarker efficacy, *PLAC4* was shown to be highly elevated in serious preterm pre-eclampsia-denoting its involvement in pre-eclampsia pathogenesis (72). As an oncogenic driver, *LINC01138* was discovered in hepatocellular carcinoma (HCC) cells that stimulated cell proliferation, tumorigenesis, tumor invasion, as well as metastasis through direct interaction with arginine methyltransferase 5 (PRMT5) (73), silencing *LINC01138* inhibited aerobic glycolysis and lactate production, thus reducing glioma cells proliferation by potentially modulating the miR-375/SP1 axis (74). The data presented herein demonstrated that these 14 LMR-lncRNAs performed critical functions in the occurrence and prognosis of LUAD. Nonetheless, there has been no investigation into their potential significance in the prognosis of patients with LUAD by considering their mechanism in lactate metabolism. Our results may open a new avenue for LUAD treatment in the future-especially for lactate-targeted combination therapy.

Notwithstanding the therapeutic relevance of our findings, some limitations need to be addressed. First, the size of the clinical sample was rather small. Second, the prognostic model needs to be evaluated in larger datasets to ensure its robustness. In addition, the investigation lacked experimental confirmation for the major modulators as well as clinical verification for the correlation between these modulators and clinical features. Furthermore, the modulatory interaction between the lncRNA and the LMR regulators is hard to deduce purely from correlation analysis, and further experimental evidence is critically required to illustrate their up- and downstream involvement. Lastly, it is necessary to investigate the potential of the genes identified as pharmaceutical targets in the near future.

## Conclusions

We discovered a new LMR-lncRNA signature associated with the prognosis of patients with LUAD, which showed a dysregulated metabolic microenvironment and might serve as a valuable prognostic indicator in patients with LUAD. The LMR-lncRNA signature may be able to differentiate survival rates based on clinical characteristics. Besides, the signature was correlated with TIICs and ICB targets. The present study provided a feasible technique for personalized risk classification of patients with LUAD and assessment of responsiveness to ICB immunotherapy, which might be clinically useful in the future. Lastly, the 14 LMR-lncRNAs highlighted by our signature could become treatment targets for LUAD.

## Data Availability Statement

The datasets presented in this study can be found in online repositories. The names of the repository/repositories and accession number(s) can be found in the article/**Supplementary Material**.

## Ethics Statement

This study was exempt from approval by the Ethics Committee of Nanfang Hospital, Southern Medical University because all data analyzed in the current study were downloaded from public databases of the TCGA (https://tcga-data.nci.nih.gov/tcga/) and GEO (https://www.ncbi.nlm.nih.gov/geo/). We simply reviewed gene expression files and corresponding clinicopathological information of patients without impeding their health and privacy disclosure.

## Author Contributions

LL, SM, and LPL designed and conducted the study. GM and DD performed the validation. LL, SM, and RC performed data extraction, quality assessment, and data analysis. SM, GM, and LPL contributed equally to this work. LL, RC, and SM wrote the manuscript. XL helped to improve the study. All authors read and approved the submitted version.

## Funding

This work was supported by grants from the Postdoctoral Research Foundation of China (No.2021M700065), and the Guangdong Basic and Applied Basic Research Foundation (2021A1515110216).

## Conflict of Interest

The authors declare that the research was conducted in the absence of any commercial or financial relationships that could be construed as a potential conflict of interest.

## Publisher’s Note

All claims expressed in this article are solely those of the authors and do not necessarily represent those of their affiliated organizations, or those of the publisher, the editors and the reviewers. Any product that may be evaluated in this article, or claim that may be made by its manufacturer, is not guaranteed or endorsed by the publisher.

## Supplementary Material

Supplementary Figure 1 Heatmap of immune infiltration in low- and high-risk groups based on TIMER, ESTIMATE, ssGSEA, QUANTISEQ, MCP counter, EPIC, and CIBERSORT.

Supplementary Figure 2 Relationship between the LMR-lncRNA signature and tumor immune dysfunction, exclusion score, immunotherapy, chemotherapy, and targeted therapy. **(A)** Distribution of TIDE scores in high-risk subtype of LUAD versus low-risk subtype of LUAD. **(B)** T cell exclusion score analysis. **(C)** T-cell dysfunction score analysis. **(D)** The correlation between anti-CTLA-4 treatment and riskscore.

Supplementary Figure 3 Relationship between The LMR-lncRNA Signature and Chemotherapeutics. **(A–F)** The half-maximal inhibitory concentration (IC50) of six common targeted therapeutics and chemotherapeutics.

## Abbreviations

LUAD, lung adenocarcinoma; ALK, anaplastic lymphoma kinase; AUC, area under the curve; ceRNA, competitive endogenous RNA; CRC, colorectal cancer; EGFR, epidermal growth factor receptor; FC, fold-change; FDR, false discovery rate; GC, gastric cancer; GEO, Gene Expression Omnibus; GO, Gene Ontology; GRCh38, Genome Reference Consortium Human Build 38; GSEA, gene set enrichment analysis; HCC, hepatocellular carcinoma; ICB, immune checkpoint blockade; KEGG, Kyoto Encyclopedia of Genes and Genomes; KM, Kaplan-Meier; KRAS, Kirsten rat sarcoma viral oncogene; LMR, lactate metabolism-related; lncRNAs, long non-coding RNAs; LUAD, lung adenocarcinoma; NSCLC, non-small cell lung cancer; OS, overall survival; PRMT5, arginine methyltransferase 5; RNA-seq, RNA sequencing; ROC, receiver operating characteristic; SCC, squamous cell carcinoma; SCLC, small cell lung cancer; ssGSEA, single-sample GSEA; TCGA, The Cancer Genome Atlas; TIICs, tumor-infiltrating immune cells; TKIs, tyrosine kinase inhibitors; TME, tumor microenvironment.
