# Supplementary figures and images for: Development and Validation of Lactate Metabolism-Related lncRNA Signature as a Prognostic Model for Lung Adenocarcinoma
Source: Front Endocrinol (Lausanne). 2022 Mar 29;13:829175. doi: 10.3389/fendo.2022.829175 (PMC9004472; doi:10.3389/fendo.2022.829175)

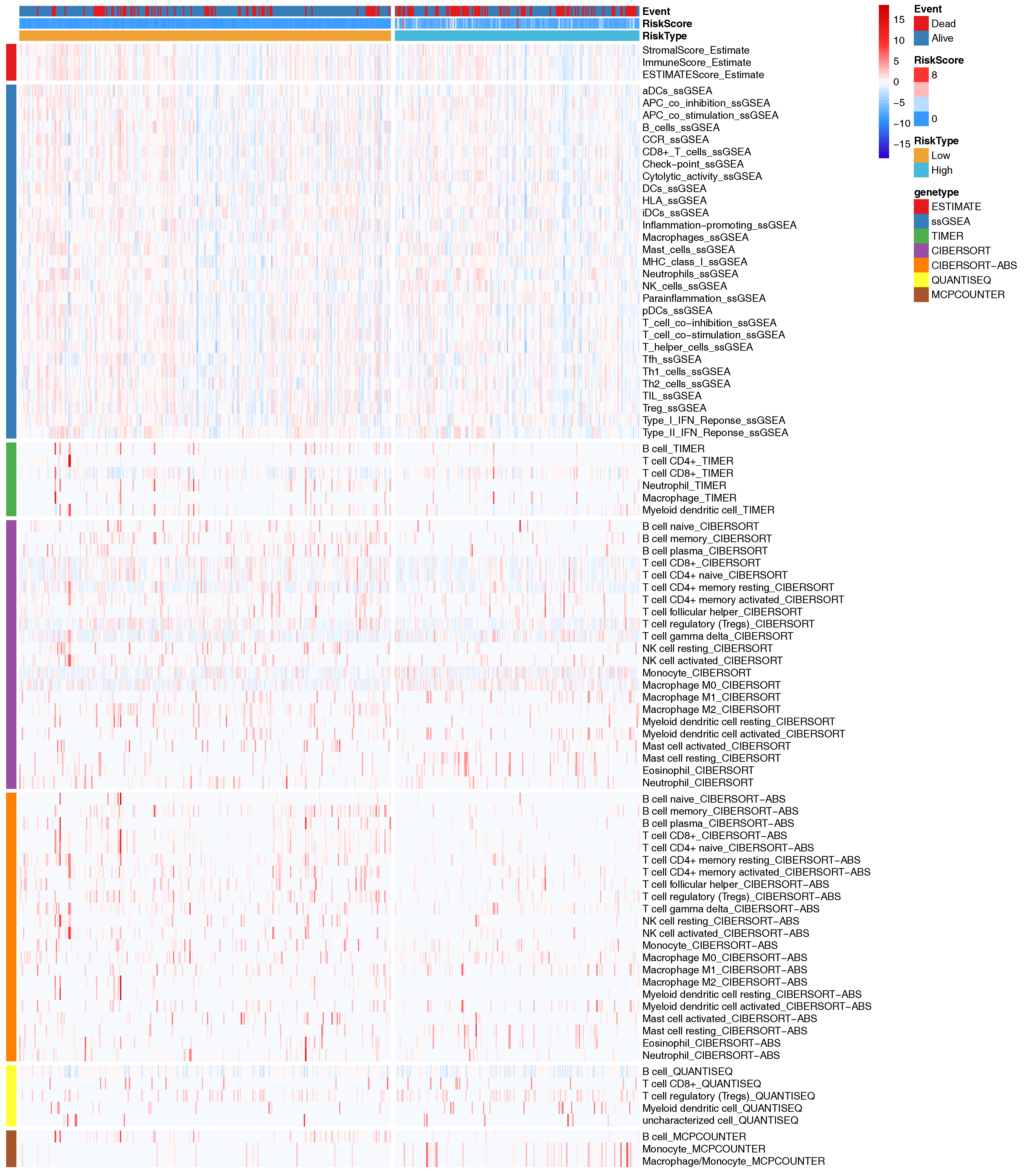

Supplement: Supplementary Figure 1 — Heatmap of immune infiltration in low- and high-risk groups based on TIMER, ESTIMATE, ssGSEA, QUANTISEQ, MCP counter, EPIC, and CIBERSORT. [file Image_1.tif]

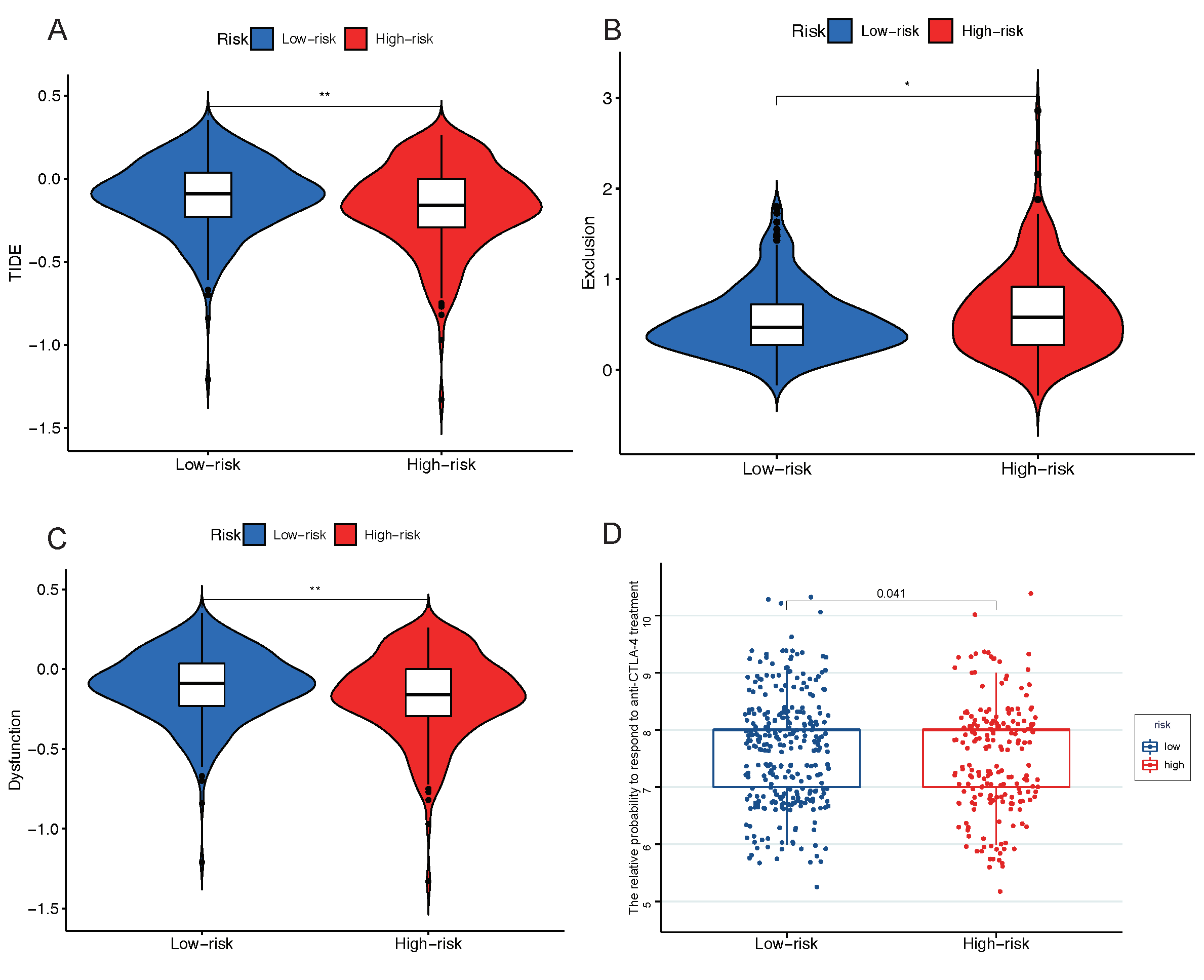

Supplement: Supplementary Figure 2 — Relationship between the LMR-lncRNA signature and tumor immune dysfunction, exclusion score, immunotherapy, chemotherapy, and targeted therapy. (A) Distribution of TIDE scores in high-risk subtype of LUAD versus low-risk subtype of LUAD. (B) T cell exclusion score analysis. (C) T-cell dysfunction score analysis. (D) The correlation between anti-CTLA-4 treatment and riskscore. [file Image_2.tif]

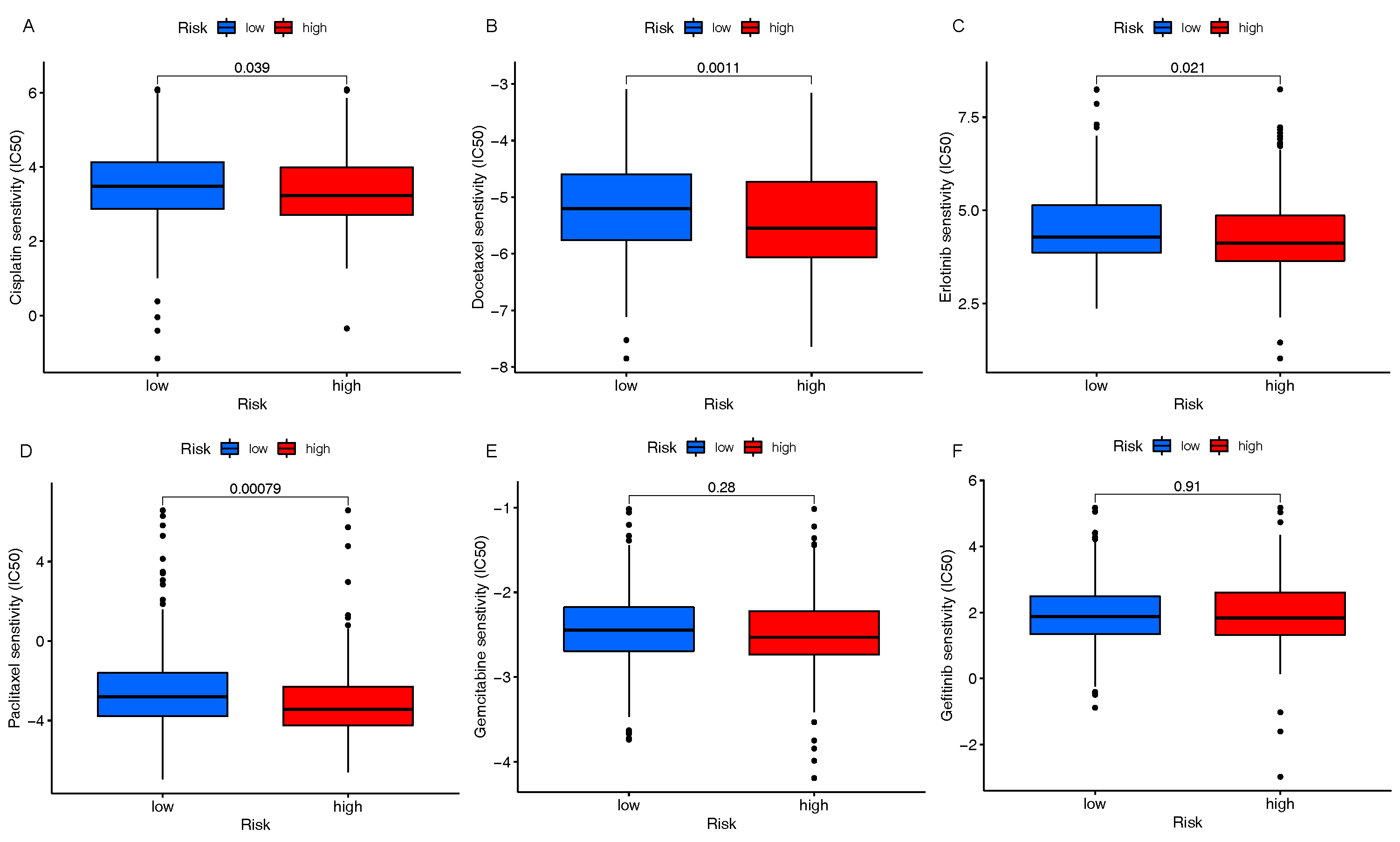

Supplement: Supplementary Figure 3 — Relationship between The LMR-lncRNA Signature and Chemotherapeutics. (A–F) The half-maximal inhibitory concentration (IC50) of six common targeted therapeutics and chemotherapeutics. [file Image_3.tiff]
